# Frequency-specific coupling in fronto-parieto-occipital cortical circuits underlie active tactile discrimination

**DOI:** 10.1038/s41598-019-41516-3

**Published:** 2019-03-25

**Authors:** Carolina Kunicki, Renan C. Moioli, Miguel Pais-Vieira, André Salles Cunha Peres, Edgard Morya, Miguel A. L. Nicolelis

**Affiliations:** 1 Edmond and Lily Safra International Institute of Neuroscience, Santos Dumont Institute, Macaíba, 59280-000 Brazil; 2 Centro de Investigação Interdisciplinar em Saúde, Instituto de Ciências da Saúde, Universidade Católica Portuguesa, Porto, 4169-005 Portugal; 3 Life and Health Sciences Research Institute, School of Medicine, University of Minho, Braga, 4710-057 Portugal; 4 Department of Neurobiology, Duke University, Durham, NC 27710 USA; 5 Department of Biomedical Engineering, Duke University, Durham, NC 27710 USA; 6 Department of Psychology and Neuroscience, Duke University, Durham, NC 27710 USA; 7 Duke Center for Neuroengineering, Duke University, Durham, NC 27710 USA; 8 Digital Metropolis Institute, Federal University of Rio Grande do Norte, Natal, 59078-970 Brazil

## Abstract

Processing of tactile sensory information in rodents is critically dependent on the communication between the primary somatosensory cortex (S1) and higher-order integrative cortical areas. Here, we have simultaneously characterized single-unit activity and local field potential (LFP) dynamics in the S1, primary visual cortex (V1), anterior cingulate cortex (ACC), posterior parietal cortex (PPC), while freely moving rats performed an active tactile discrimination task. Simultaneous single unit recordings from all these cortical regions revealed statistically significant neuronal firing rate modulations during all task phases (anticipatory, discrimination, response, and reward). Meanwhile, phase analysis of pairwise LFP recordings revealed the occurrence of long-range synchronization across the sampled fronto-parieto-occipital cortical areas during tactile sampling. Causal analysis of the same pairwise recorded LFPs demonstrated the occurrence of complex dynamic interactions between cortical areas throughout the fronto-parietal-occipital loop. These interactions changed significantly between cortical regions as a function of frequencies (i.e. beta, theta and gamma) and according to the different phases of the behavioral task. Overall, these findings indicate that active tactile discrimination by rats is characterized by much more widespread and dynamic complex interactions within the fronto-parieto-occipital cortex than previously anticipated.

## Introduction

It is commonly assumed that tactile sensory processing is transmitted from the periphery to the cortex through parallel feedforward pathways, which ran through well-defined neuronal aggregates, grouped into rows and arcs, in the brainstem (barrelets), thalamus (barreloids), and somatosensory (barrel) cortex, which altogether generate a multi-level isomorphic representation of the whiskers of the snout of most rodents^1,2^. According to this classic model, the primary somatosensory cortex (S1) is a specialized cortical area dedicated to the processing of somatic information only. However, recent studies have revealed an intricate network of structural and functional connections linking primary sensory areas to higher-order regions^3,4^. Moreover, processing of tactile information is influenced by multiple thalamo-cortical, cortico-cortical^5–7^ and subcortical^8^ loops that reflect higher order influences related to tactile learning^9,10^, behavioral state^11,12^, motor activity^13,14^, reward expectation^15,16^ and attention^17^. Although the involvement of higher-order integrative cortical areas in somatosensory processing is broadly reported, the neural mechanisms underlying the flow of information between S1 rand high-order areas remains, for the most part, totally unclear.

According to electrophysiological^18–20^ and functional imaging studies^21,22^, the anterior cingulate cortex (ACC) is capable of coordinating top-down responses. ACC is anatomically and functionally interconnected with other areas, including somatosensory, motor and subcortical areas^23,24^, and it has been implicated in functions such as long-term memory^25,26^, cost-benefit decision-making^27,28^ and attention^29,30^. These higher-order functions are thought to be sustained by ACC during the interaction with sensory and association areas^31,32^. In addition, studies have consistently shown that ACC and the posterior parietal cortex (PPC) play an important role in modulating the processing tactile information in the somatosensory cortex^33,34^. For example, ACC stimulation increases tactile responses and alters basal activity in the ventrobasal region of the thalamus^35^. Likewise, PPC injuries can lead to deficities in a tactile discrimination task^36,37^.

Here, we hypothesized that active tactile discrimination may be mediated by widespread and dynamically complex bidirectional communication between S1 and other cortical areas located in the frontal, parietal, and even the occipital cortex (e.g. primary visual cortex). According to this hypothesis, both so called lower and higher order cortical areas interact differently depending on the animal’s behavior and the different phases of a tactile discrimination task. In order to test this central hypothesis, we first characterized single-unit responses in the ACC, PPC, S1 and V1 neurons during different phases of an active tactile discrimination task to analyze the type of task-related neuronal modulations observed throughout a frontal-parietal-occipital cortical circuit. Next, using multi-site LFP recordings, we assessed the synchronization dynamics and directional information flow in the same fronto-parieto-occipital loop, as a way to identify the presence of a hallmark of cross-modal interactions across a vast cortical territory^38,39^. Overall, our results revealed the existence of frequency-specific, and dynamically complex cortical interactions taking place throughout the fronto-parieto-occipital circuit during active tactile discrimination by rats.

## Results

### Behavioral responses

**Figure 1:**
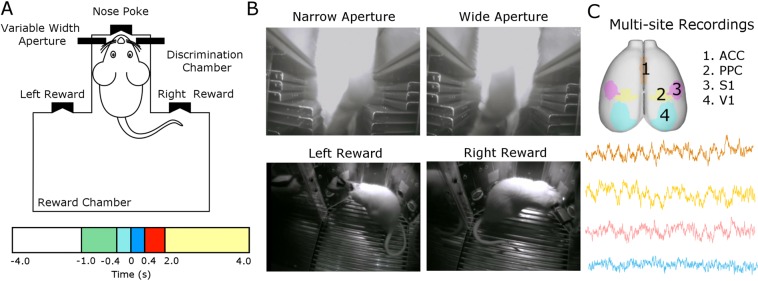
Experimental design of the tactile discrimination task and recorded cortical areas. (**A**) Schematic representation of the behavioral apparatus. Briefly, the apparatus consists of a box with two compartments: discrimination and reward chambers separated by sliding door. The discrimination chamber contains a variable width aperture (wide or narrow) and a central nose poke. The other compartment, reward chamber, contains two reward ports (left and right). The behavioral task was divided in phases according to the animal’s behavior and it localization in relation to the box [anticipatory: −1.0 to −0.4 s (green), discrimination 1: −0.4 to 0 s (light blue), discrimination 2: 0 to 0.4 s (dark blue), response: 0.4 to 2.0 s (red) and reward: 2.0 to 4.0 s (yellow)]. The instant t = 0 s is defined as the moment that the animal reaches the central nose poke, and thus, experience the maximum vibrissae deflection. (**B**) The rats were trained to discriminate between a narrow (52 mm) versus wide (85 mm) aperture using only their mystical vibrissae to receive a water reward in the left or right rewards, respectively. (**C**) Unit activity and local field potential were recorded from four cortical regions (anterior cingulate cortex [ACC], posterior parietal cortex [PCC], primary somatosensory cortex [S1] and visual cortex [V1]) during the active tactile discrimination task.

A total of 9 Long-Evans rats were implanted with microelectrode arrays. While the animals performed an active tactile discrimination task^10,40^ (see Fig. 1), single-unit and local field potentials (LFP) were simultaneously obtained in unilateral ACC, PPC, S1, and V1. On average, the animals performed 181.33 ± 46.58 trials and had a performance of 80.11% ± 9.21 (average correct trials), in line with previous results obtained in this task^6^. A total of 673 cortical neuronal units were recorded in the four regions implanted over 9 sessions of the behavioral task (one session per animal). The proportion of units registered in each region were as follows: 24% in ACC (n = 164 units), 24% in PPC (n = 165 units), 30% in V1 (n = 202 units) and 22% in S1 (n = 142 units).

### Neuronal firing modulations occur throughout the sampled frontal-parieto-occipital circuit during the whole trial

The recorded single-unit firing modulations in ACC, PPC, S1 and V1 were used to calculate proportion, magnitude, duration and type of firing modulations (increase, decrease and multiphasic) during the behavioral task. Statistically significant modulations in the firing rate were observed in a large proportion of the neurons recorded in all the sampled cortical areas (Table 1). Specifically, we found patterns characterized by increased, decreased and multiphasic neuronal activity that varied between different regions. In each region, firing modulations were observed in 72% of the units recorded in ACC, 81% of units registered in PPC, 60% of units registered in V1, and 66% of the units registered in S1. Magnitude and duration of increased and decreased firing responses per cortical area are presented in Table 1. Multiphasic neurons were found in all studied regions, with the highest proportion found in V1 (38%) when compared to S1 (33%), ACC (27%) and PPC (17%).

**Table 1:** Neuronal firing modulation patterns by cortical area during active tactile discrimination.

Region	Modulation	Responsive			Unresponsive
		Increased	Decreased	Multiphasic	
PFC	Fraction	0.54 ± 0.02	0.18 ± 0.02	0.27 ± 0.01	0.19 ± 0.02
	Magnitude (spikes/s)	3.88 ± 0.29	2.19 ± 0.17		
	Duration (s)	0.36 ± 0.05	0.20 ± 0.04		
PPC	Fraction	0.45 ± 0.03	0.36 ± 0.04	0.17 ± 0.02	0.31 ± 0.02
	Magnitude (spikes/s)	2.93 ± 0.20	2.40 ± 0.15		
	Duration (s)	0.26 ± 0.05	0.28 ± 0.05		
S1	Fraction	0.36 ± 0.01	0.30 ± 0.02	0.33 ± 0.02	0.11 ± 0.02
	Magnitude (spikes/s)	4.27 ± 0.45	2.70 ± 0.22		
	Duration (s)	0.49 ± 0.08	0.20 ± 0.02		
V1	Fraction	0.30 ± 0.02	0.30 ± 0.03	0.38 ± 0.03	0.13 ± 0.02
	Magnitude (spikes/s)	4.83 ± 0.58	3.46 ± 0.30		
	Duration (s)	0.32 ± 0.03	0.18 ± 0.02		

Neuronal activity was labeled as “increased” or “decreased”, if the neuronal firing rate increased or decreased, respectively, considering the baseline period, or “multiphasic”, if a given neuron showed both increased and decreased modulation. If no modulation was found, neurons were labelled “unresponsive”. The table shows the fraction of each neuron type, the response magnitude (the average difference in firing rate between the significant firing modulation period and the baseline) and the neuronal response duration (the average time for which the significant firing modulation was sustained).

**Figure 2:**
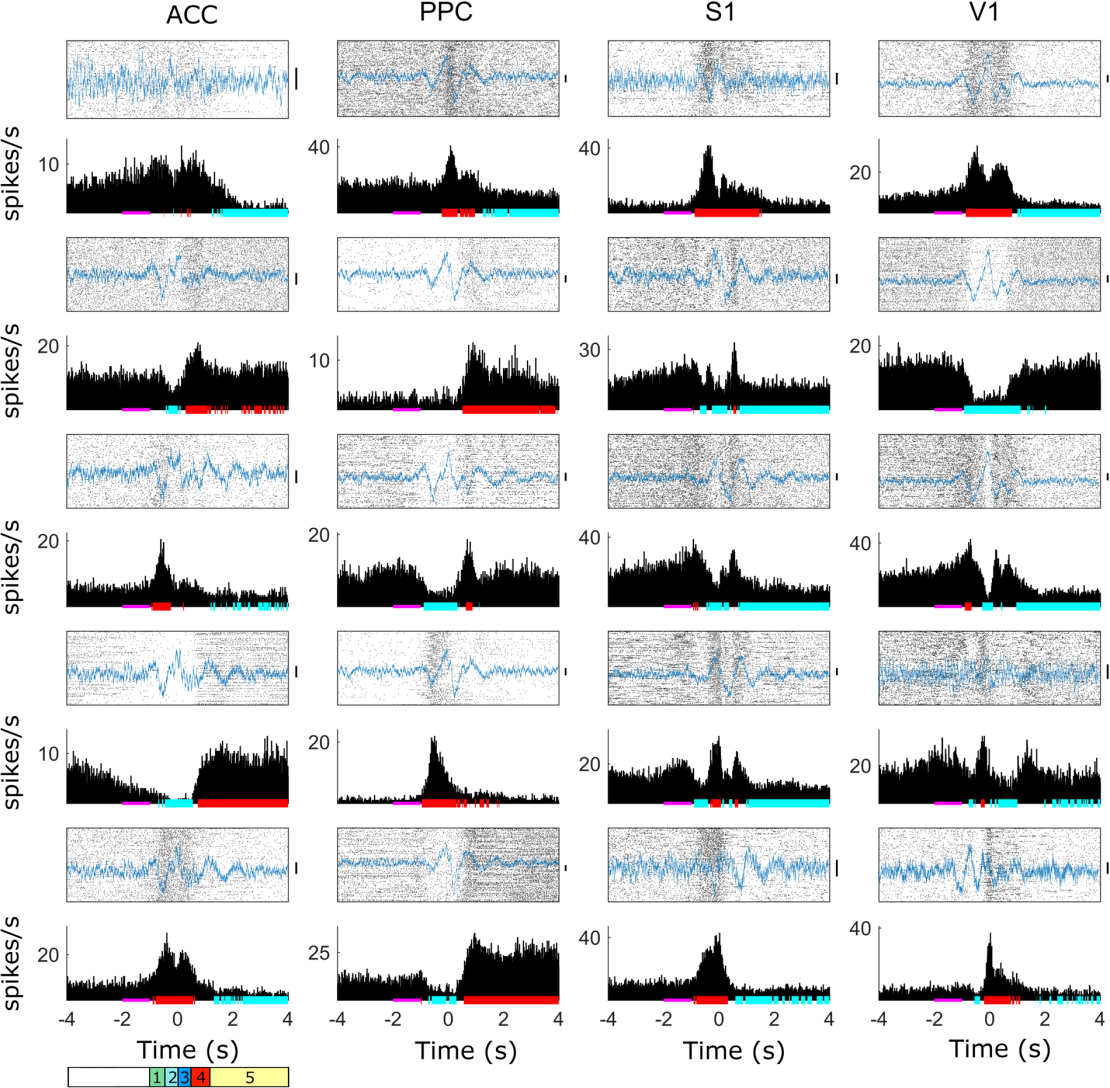
Single peri-event histogram recorded during active tactile discrimination task in the ACC, PPC, S1 and V1. Periods of increase and decrease in neuronal activity occurred during all task stages [anticipatory (green 1), discrimination 1 (light blue 2), discrimination 2 (dark blue 3), response (red 4) and reward (yellow 5) in all recorded cortical areas. The instant t = 0 s is defined as the moment that the animal reaches the central nose poke. Solid pink bar along the x-axis indicates baseline period. Increase and decrease of statistically significant neuronal activity are indicated by red and blue lines, respectively. Examples trace showing raw LFP of each brain region are shown in blue superimposed each raster plot. Black vertical bars next to each LFP indicate the 0.2 mV scale.

**Figure 3:**
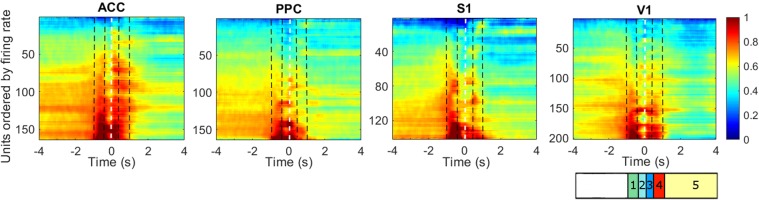
Representation of neuronal activity recorded simultaneously across the frontal-parietal-occipital cortex during execution of an active tactile discrimination task. Each figure shows the peristimulus time histograms (PSTH) from all neurons recorded in different structures. From left to right: ACC (164 units), PPC (165 units), S1 (142 units), and V1 (202 units). Each row in each figure represents the activity of a neuron normalized to its average firing rate during the baseline period ([−3, −1] s). Each color represents a variation in the firing rate. Increase is in red; decrease is in deep blue. Units were ordered by the average firing rate in −1.5 to −0.5 s. The dotted white vertical line marks the center nose poke (CNP) task period (Time 0). There were neurons with increased firing rate immediately before the whiskers contacted with CNP in all recorded regions. The vertical black dashed lines divide the figures in the task periods: anticipatory: −1.0 to −0.4 s (green 1), discrimination 1: −0.4 to 0 s (light blue 2), discrimination 2: 0 to 0.4 s (dark blue 3), response: 0.4 to 2 s (red 4) and reward: 2 to 4 s (yellow 5).

In all sampled cortical areas, neuronal firing rate modulations were not restricted to a specific period (see Fig. 2). Instead, they occurred throughout all task stages (anticipation, discrimination, response, and reward; also see methods for a detailed description of each task phase). Anticipatory neuronal firing modulations were observed in all regions (ACC, PPC, V1 and S1). These modulations often occurred a few hundred milliseconds before the animal’s vibrissae touched the tactile discrimination bar and continued for a few hundred milliseconds after the tactile stimulus. Examples of such anticipatory modulations in different cortical regions can be observed in the peri-stimulus histograms (PSTH) (Fig. 2). Overall, during anticipatory period we documented the presence of an increase in neuronal firing in all cortical areas recorded simultaneously (Fig. 3). However, decreased responses can also be observed in the same areas. A predominance of increased-decreased-increased responses was observed in the discrimination period. These complex firing modulations were characterized by an increase in the firing rate at the beginning of the discrimination, followed by a brief decrease, and then an increase at the end of the discrimination period. The response period was marked by the presence of an increase in neuronal firing modulation, mainly in ACC. At this time, a concurrent reduction in firing rate was observed in S1 and V1. Interestingly, during the reward period, we documented the occurrence of a substantial decrease in neuronal firing in all regions, which was higher in S1 and V1 (Fig. 3).

### Generalized phase locking with V1 before tactile sampling

**Figure 4:**
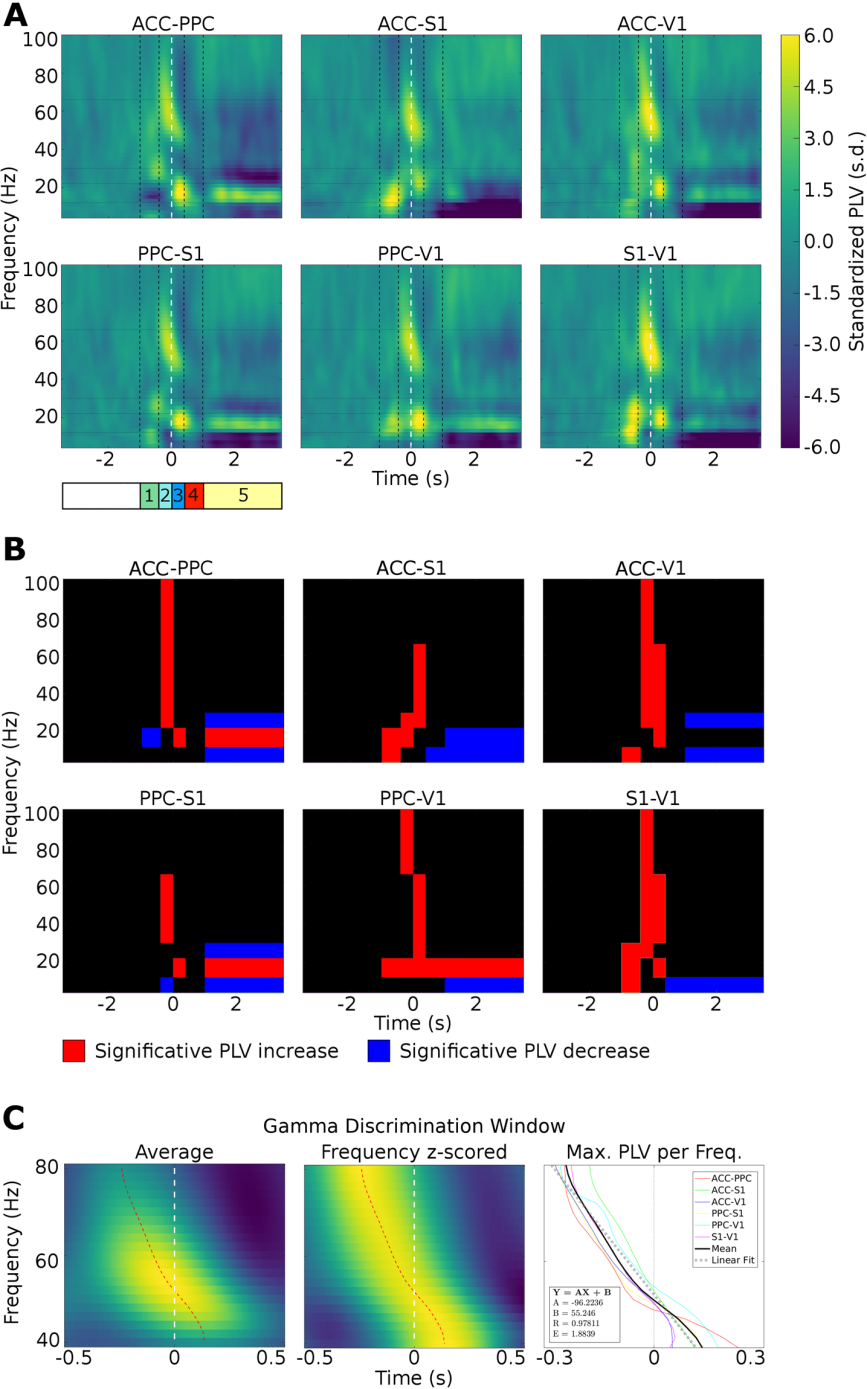
Phase synchronization increases during whisker discrimination task in theta (4–12 Hz), beta 1 (13–21 Hz), beta 2 (22–30 Hz), gamma 1 (31–65 Hz) and gamma 2 (66 to 100 Hz) frequency bands. (**A**) Average time-frequency charts of the phase synchronization changes obtained during the task, for each pair of recorded regions (ACC – PPC; ACC – S1; ACC – V1; PPC – S1; PPC – V1 and S1 – V1). Each chart represents the z-score (to the respective [−3 −1] baseline period) average phase synchronization across all pairs of electrodes and subjects. The dotted white vertical line marks the CNP task period. The vertical black dashed lines divide the figures in the task periods. The following behavioral epochs were defined: anticipatory from −1.0 to −0.4 s (green 1); discrimination 1 from −0.4 to 0 s (light blue 2); discrimination 2 from 0 to 0.4 s (dark blue 3); response from 0.4 to 2.0 s (red 4) and reward from 2.0 to 4.0 s (yellow 5). Major changes are observed in theta (4–12 Hz), beta (13–30 Hz), low gamma (30–70 Hz) and high gamma frequency bands (70–100 Hz). Note that there are specific time-frequency values where cortical regions become synchronized across the task. (**B**) Significance of phase locking value during each epoch of the tactile discrimination task. The plot with the red bar indicates significative PLV increase and blue bar indicates significative PLV decrease. Was considered as significant values those higher than baseline mean plus two times its standard deviation. (**C**) Left: Average gamma phase synchronization in a window of −0.5 to 0.5 s from 40 to 80 Hz for all pairs of regions recorded in this study. The dotted red curve represents the instant of maximum phase synchronization for each frequency. Centre: same as before, but frequency Z-scored. Right: pairwise maximum frequency z-scored. Linear fit is related to the average curve of the maximum PLV. The angular coefficient of the linear regression was −96. In other words, at every 0.01 s peak PLV was observed 1 Hz down from the current frequency value.

In order to evaluate phase interactions throughout the frontal-parietal-occipital circuit, we conducted a phase synchronization analysis according to Lachaux *et al*.^41^. This measure has been widely proposed as being able to infer neural communication^42,43^. Figure 4A,B shows the pairwise mean phase-locking, for all pairwise combinations of cortical areas, over time for all animals, for each period (anticipation, discrimination, response and reward) in relation to baseline (from −3 to −1 s).

Overall, the pattern of intercortical area synchronization markedly changed between the different phases of the tactile discrimination task. As depicted in Fig. 4A,B (phase locking), increased synchrony was found during the anticipatory period (starting at −1.0 seconds) in the theta (4–12 Hz) and beta bands (13–30 Hz) in most pairs of the analyzed regions. This phase locking rise occurred between V1 and all other areas (V1-S1, V1-PPC, V1-ACC). Thus, even though rats were performing the task in the dark, V1 presented phase locking to all other areas even before the tactile stimulus started (−1.0 to −0.4 seconds). During this period, we also observed an overall decrease in PLV for ACC-PPC in the beta 1 band (13–21 Hz).

### Tactile discrimination is associated with phase locking rise across all areas

During the discrimination periods, we found prominent phase synchronization distributed across all studied regions. As indicated previously, the discrimination was divided into two periods, Discrimination 1 and 2. In both discrimination periods, an increase in synchrony in the beta (13–30 Hz) and gamma bands (31–100 Hz) were found in most pairs of cortical areas. Moreover, an increase in synchrony in the beta 1 (13–21 Hz) band was found for discrimination 2 in all pairs of analyzed regions (Fig. 4A,B). The sole exception to this was ACC-S1, where an increase was found in beta 2 (22–30 Hz).

Interestingly, we found changes in the maximum PLV value over time by frequency in the gamma band (40–80 Hz) in all pairs of regions studied during the tactile discrimination (−0.4 s to 0.4 s): at every 0.01 s peak PLV was observed 1 Hz down from the current frequency value (see Fig. 4C).

### Generalized phase locking with PPC during response and reward periods

In contrast to discrimination periods 1 and 2, both the task response and reward periods were marked by an overall decrease in synchrony across regions and frequency bands. However, as depicted in Fig. 4, PPC remained intensely phase locked to all other regions in a very narrow beta band (13–21 Hz). Also noteworthy, the phase locking between PPC-V1, which had already started in the anticipatory period (−1.0 seconds) in this particular band, remained until the end of the trial. Meanwhile, phase locking of PPC-ACC and PPC-S1 was briefly interrupted during the initial stage of the response period (0.4 to 2.0 seconds). This roughly corresponds to the moment where the rat has already sampled the aperture and was starting to nose poking the reward port.

In summary, our analysis of phase locking revealed that generalized phase locking with V1 occurred before tactile sampling (but also between ACC-S1), followed by massive synchronization across all pairs of cortical regions, in multiple frequency bands, during tactile sampling. Lastly, an extended period of overall phase locking with the PPC, that lasted until the end of the trial, occurred within a narrow frequency band (13–21 Hz). Interestingly, a greater level of desynchronization, predominantly in theta and beta 2 bands, was observed during the reward period.

### Granger Causality

**Figure 5:**
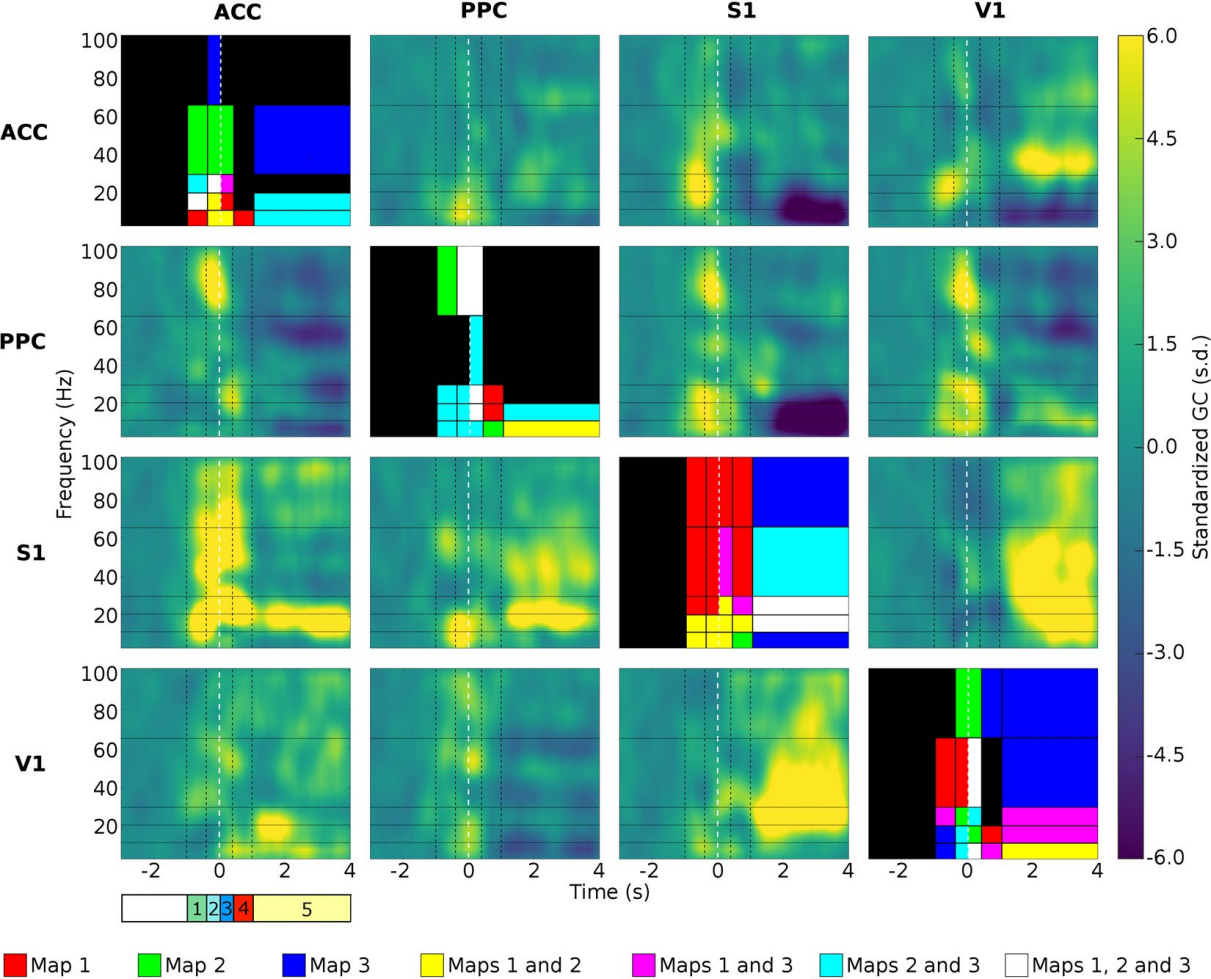
Grid of spectral Granger causality maps in the frontal-parietal-occipital loop. The colormap is presented in baseline standard deviation units (how many times a GC is greater or lesser than the baseline standard deviation). The lines in figure grid represent the cortical structures were the information was originated, while the columns represent the target structures (GC direction is defined from the structure that originates the information to target structure). The instant t = 0 s (dashed white line) is defined as the instant that the animal reaches the central nose poke, and thus, experience the maximum vibrissae deflection. The vertical black dashed lines divide the figures in the task periods (anticipatory – green 1, discrimination 1 – light blue 2, discrimination 2 – dark blue 3, response – red 4 and reward – yellow 5), and the horizontal lines divide the figure in frequency bands (theta 4–12 Hz, beta 1 [13–21 Hz], beta 2 [22–30 Hz], gamma 1 [31–65 Hz] and gamma 2 [66 to 100 Hz]). Absolute GC variations from the baseline, higher than two times the baseline standard deviation, were considered significant. The figures in the grid diagonal show the significant regions of the spectral GC maps. Each diagonal figure brings information about the three other GC maps at the same line. The colored regions indicate the significant map regions, where red represents the first map that appears in that line grid (left to right), green the second and blue the third. If a region was significant in more than one map, the color of the maps should be overlayed (maps 1 and 2 = yellow; maps 1 and 3 = magenta; maps 2 and 3 = cyan; maps 1, 2 and 3 = white). The maps reveal both top-down and bottom-up directed influence, which was stronger and time-frequency specific during active tactile discrimination task. Spectral GC values were estimated with a 150 ms window running in 10 ms time steps.

To determine to which extent the changes observed in the activity of single units and the overall patterns of cortical phase locking were associated with causal changes in neuronal processing, we calculated the Granger causality between pairs of recorded cortical areas^44^. Significant Granger causality modulation was used to infer the direction of information flow between cortical areas sampled within the frontal-parietal-occipital circuit during active tactile discrimination. Overall, we observed significant two-way Granger causality between sensory and higher order cortical areas during the different task phases (Fig. 5). We also found causal influence between primary sensory areas (V1 to S1 and S1 to V1), as well as between higher order areas (ACC to PPC and PPC to ACC). This causal influence did not simultaneously occur among all cortical areas. Instead, it was restricted to specific pairs of cortical regions with the predominant frequency bands varying at specific task phases. Both transient (100 to 200 ms) as well as longer (1 to 2 s) Granger causality significant periods were observed. What follows is a more detailed description of these results.

### S1 and V1 drive the ACC and PPC throughout the task

As depicted in Fig. 5, at −1.0 seconds, a period in which rats were typically waiting to start a trial, Granger causality analysis demonstrated that most cortical areas drove the activity of other cortical regions already. For example, both S1 and PPC started to influence ACC activity at different frequency ranges during this period. S1 continued to influence the ACC until the end of the task (see Fig. 5). At this point (−1.0 second), S1 also started to influence PPC activity and continued to do so throughout the remaining of the task. In addition, the ACC began to influence S1 and V1 neuronal activity at −1.0 seconds. It is noteworthy that, like the pattern observed in phase locking, this early increase in Granger causality often included multiple frequency bands (see Fig. 4).

### Tactile discrimination is associated with widespread causal interactions between cortical areas

During the period of discrimination, all cortical areas were, to some extent, driving the activity other areas, and they did so generally across multiple frequency bands. The exception to this was the influence exerted from S1 to V1 that, during this period, occurred for a very small period of time (0 to 0.4 seconds) in a rather restrict frequency band (between 31–65 Hz). Despite this difference, during the tactile discrimination period, the generalized pattern of cortical areas driving each other matched the results observed with the phase locking analysis.

Immediately after tactile sampling (0.4 to 2.0 seconds), ACC stopped driving S1 and V1, V1 stopped driving the PPC, and all other three cortical areas stopped driving V1 (ACC, PPC and S1; see Fig. 5). Meanwhile, V1 remained influencing the ACC and S1. In fact, V1 began to influence both ACC and S1 neuronal activity at −1.0 seconds and remained doing so until the end of the trial (even if through different frequency ranges at each task phase). Thus, as observed in phase locking analysis, while tactile discrimination was characterized by a massive interactions between multiple cortical areas, the period immediately after was characterized by a generalized reduction in interaction between recorded cortical sites. As this latter response period ended and the rat received a reward (>2.0 seconds), apart from the aforementioned S1 and V1 influence over other areas, two other major pairwise cortical interactions were enhanced. First, the ACC remained driving V1 until the end of the trial. Likewise, the PPC continued to influence the V1 until the end of the trial within a narrow frequency band (beta 1, 13–21 Hz), in a pattern somewhat similar to the results observed with phase locking analysis (compare with Fig. 4).

**Figure 6:**
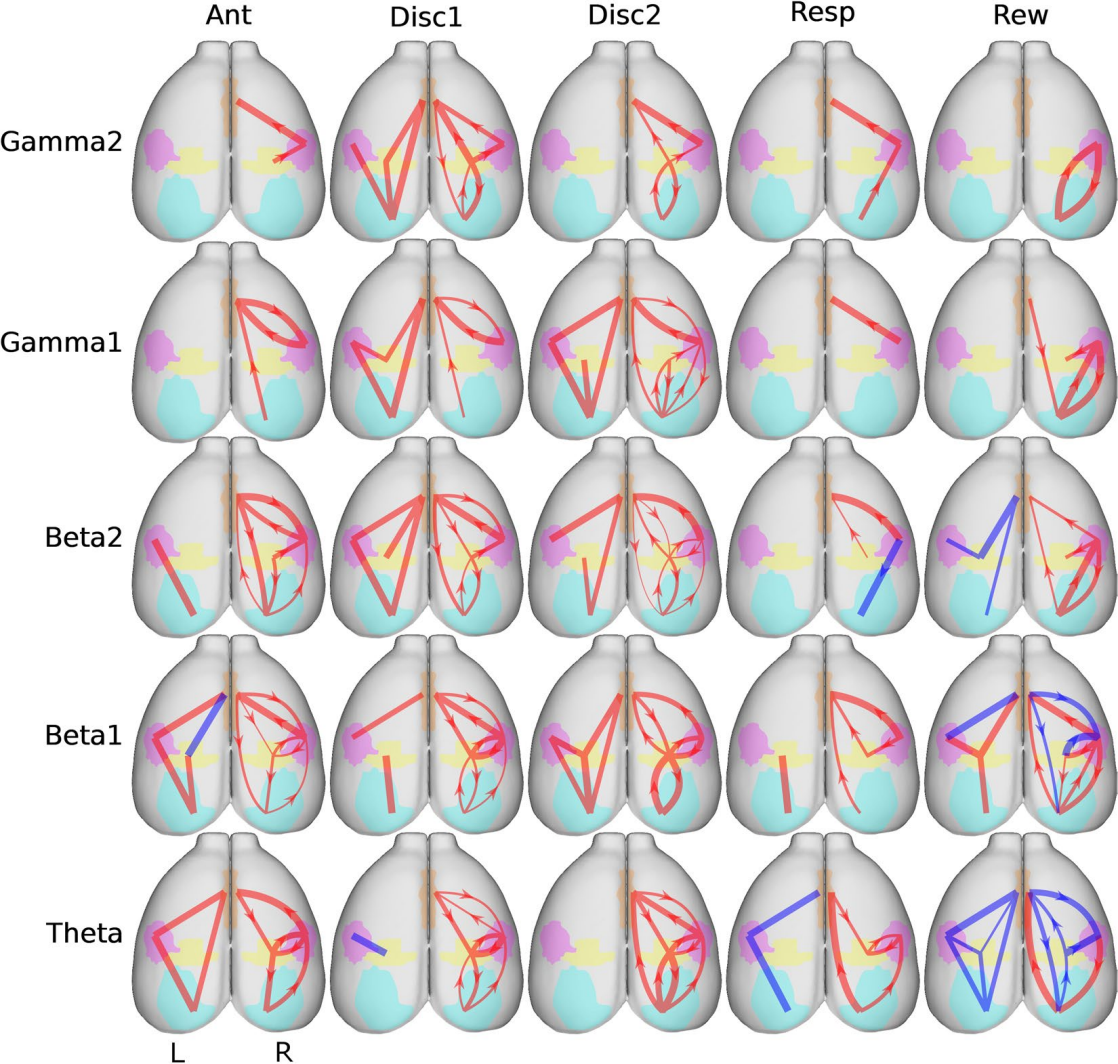
Summary of granger causality findings supporting distributed processing during an active tactile discrimination task. The left hemisphere represents the PLV findings (L) while the right hemisphere represents the GC results (R). The PLV was represented in the left hemisphere as didactic resource. Note that both phase lock and Granger causality analysis are the result of recordings performed on the right hemisphere. The thickness of the edges indicates the strength of the synchrony (PLV) and information flow or G-connectivity (GC) between the structures. The red edges indicate a rise while the blue edges indicate a decrease in synchrony or G-connectivity between the brain structures. In the GC graphs (right hemisphere), the arrows indicate the direction of the information flow. The ACC is represented by the orange region, PPC yellow, S1 pink, and V1 light blue. The graphs suggest that both primary sensory areas and higher order areas can drive responses depending on the animal behavior.

A comparison of the results obtained with phase lock and Granger causality are presented in Fig. 6. Both phase lock and Granger causality indicate a period of massive synchronization and information transfer occurring during the tactile sampling, followed by a general decrease immediately after. In addition, both analyses indicate that each particular period of the task is characterized by very specific interactions between cortical areas that may or may not be coincident for both analysis (i.e. phase lock did not imply Granger causality).

## Discussion

In this study, we simultaneously recorded electrical activity from cortical neurons distributed across the frontal-parietal-occipital loop while rats performed an active tactile discrimination task. Analysis of these concurrent cortical recordings revealed the occurrence of statistically significant neuronal firing rate modulations throughout the ACC, PPC, V1 and S1, during multiple phases of the task. We have also found that multiple cortical areas tend to present phase locked LFP activity in the period surrounding sampling of the tactile discriminanda. Granger causality analysis revealed multiple task-related interactions occurring between the cortical areas sampled.

A wide range of firing rate modulations were evoked in all cortical areas including increased, decreases and multiphasic responses. These data are consistent with results shown by other authors who utilized the same task in S1^5,6,40^ and V1^45^. We also found extensive neuronal firing rate modulations in the ACC and PPC, which demonstrate the clear involvement of these cortical areas in tactile discrimination according to electrophysiological^35,46,47^ and functional imaging studies^48,49^. The presence of marked modulations in the neuronal firing rate in all task phases (anticipation, discrimination, response and reward) also suggest that even such a simple tactile discrimination task requires the involvement of vast cortical circuits to be performed properly. This key finding suggests that, during an active exploration, such as the one required to perform the task employed in our study, processes such as attention and sensorimotor integration are essential and likely require the recruiting of vastly distributed cortical networks^34,50^ to allow animals to perform the task effectively. In line with this finding, other studies have shown that primary sensory areas are involved in complex cognitive process such as reward timing^51^, reward contingency^15^, attention^52^ and that higher order areas may influence sensory processing^53,54^.

Anticipatory firing rate modulations by cortical neurons (i.e. even before the animal’s vibrissae touched the tactile discrimination bars) were found to occur in all regions investigated in this study. The presence of anticipatory activity in this particular task has been previously identified in S1^5,10,12^, the posterior parietal cortex and premotor cortex^55^, and in thalamic nuclei belonging to the rat trigeminal somatosensory system^5^. Moreover, the same study showed that M1 inactivation altered anticipatory activity in S1 and in the thalamus, suggesting that such anticipatory activity depends on generated top-down effects, partly by M1, and that it cannot be solely explained by the traditional feedforward model of the rat trigeminal system^5^. As statistically significant modulations were observed in all cortical areas during the anticipatory period, it is possible that this overall response in the frontal-parietal-occipital loop illustrates the contribution of such top-down effects throughout this cortical circuit. Moreover, it is possible that these anticipatory modulations may also influence thalamic-cortical dynamics in the trigeminal system. However, our experiment did not investigate the origin of these anticipatory modulations. Future experiments will evaluate whether ACC lesions/block, for example, can reduce anticipatory modulations in the S1 and subcortical structures belonging to the trigeminal system.

The working hypothesis tested in the present study was that active tactile discrimination require an intense dynamic information exchange between ACC, PPC, S1 and V1. To test this hypothesis, we calculated the pairwise phase synchrony between these four recorded regions, which is a measurement associated with neural communication^42,43,56^. Our results showed an intense neural synchrony between all recorded cortical areas sampled in the frontal-parietal-occipital circuit. This cortical communication dynamics changed in specific frequency bands over the course of the task phases. In general, strong synchrony was observed in the anticipation and discrimination periods in the theta/beta and beta/gamma bands, respectively. On the other hand, the response and reward periods were marked by a decrease in synchrony, predominantly in the theta/beta bands. Recent studies have suggested that the activity in each neuronal ensemble can be modulated by its own local oscillation and it is the phase difference between these distinct oscillations that may enable the selection and the processing of individual items^42,57,58^.

Previous studies support the idea that neural synchrony (coherence or phase-locking) can provide a communication link between regions^58,59^ of the same or different hierarchical levels^38,60^. Indeed, Nicolelis *et al*.^61^ have suggested that tactile information is represented in the trigeminal system in a very dynamic and spatiotemporally distributed way. Our findings suggest the existence of a cortical mechanism capable of synchronizing the activity of multiple, spatially dispersed neural structures. Such a synchronization could generate a highly attentive state that allows the animal to anticipate and, possibly, better discriminate the received tactile information. Indeed, our results have consistently shown the occurrence of distributed anticipatory activity and long-range synchrony between ACC, PPC, S1 and V1, predominantly in the anticipatory and discriminatory periods. Recently, Cicurel and Nicolelis^62^ have suggested that cortical electromagnetic activity could provide the type of long-range synchronizing signal capable of producing the type of large-scale cortical entrainment observed in our study.

It is important to emphasize that there are also areas or even pathways which may not be engaged in the task. For example, in one of the experiments by Pais-Vieira *et al*.^5^, the authors have shown that there are regions of ventral posteromedial nucleus (VPM) which are not modulated, even within the trigeminal system. Primary motor cortex (M1) inactivation altered the magnitude of the anticipatory responses in the core region of VPM, but not of the VPM head region during an active tactile discrimination task. In another paper recently published by Bieler *et al*.^63^, the authors showed that simultaneous tactile and visual bimodal stimulation in rats under light anesthesia modulates the evoked activity in VPM, but not in the dorsal lateral geniculate nucleus (dLGN). Experiments in humans with single pulse transcranial magnetic stimulation have also demonstrated the differential involvement of pathways from S1 to the middle and superior frontal gyrus during a tactile discrimination task^7^.

Another very relevant point is what would be expected of this rich dynamic interaction between brain areas in animals with different performances. Using an auditory detection task in rats, Herzog *et al*.^64^ has reported a decreased long-range frontal-parietal coherence of beta (15–30 Hz) band when the animal correctly executed the task and elevated theta or alpha coherence (4–15 Hz) when the animal failed to respond. Additionally, Grion *et al*.^65^ showed that rats with better performance and faster responses presented increased coherence between whisking rhythm and hippocampal theta oscillation during a texture discrimination task. Consistently, in humans, Wang *et al*.^66^ demonstrated greater long-range interaction between the brain networks of individuals with higher performance in reading tasks using functional connectivity analyses.

Additionally, we used Granger causality to investigate the directed information flow in the frontal-parietal-occipital loop during the active tactile discrimination task. Our results showed that both primary sensory (S1 and V1) and higher-order integrative areas (ACC and PPC) can influence each other as a function of the animal’s behavior. A G-causal link from low to higher order areas is expected based on the traditional model of the hierarchical processing of sensory information^67,68^. In contrast, driving modulations from higher to lower order areas may occur because many of the inputs to primary sensory areas do not originate from the thalamus, but rather from higher order cortical regions^3,35^. Corroborating our results, which show that top-down inputs can drive responses in primary sensory areas^69,70^, recorded V1/V2 and A1/A2 of mice stimulating feedforward or feedback afferents and observed drive responses in both cases, as well as modulatory responses^69,70^. These data are consistent with previous studies that showed driving effects of connections from area V2 to V1 in the absence of inputs from the geniculate nucleus^71^. Altogether, these findings indicate that so called higher cortical areas can drive lower structures, via feedback projections, as strongly as feedforward pathways normally do. Supporting this view, our results showed the occurrence of dynamic G-causal links between hierarchically distant cortical areas (both ACCC and PPC drove responses in primary sensory areas S1 and V1).

We also found causal influence between lower order cortical areas related to different sensory modalities (tactile and visual), as well as between higher order areas (anterior cingulate cortex and posterior parietal cortex). Multisensory integration has been extensively described in numerous species including rats^72,73^, monkeys/primates^74,75^ and humans^76,77^. Furthermore, a recent study has shown that electrical or optogenetic stimulation of V1 drives spiking in VPM^78^, which provides support for the notion that V1 neurons can influence VPM activity via cortico-cortical connections to the S1.

It is important to emphasize that our analysis revealed that causal interactions did not occur simultaneously between all cortical areas, but rather in specific frequency bands, between pairs of specific cortical regions, and during particular phases of the tactile discrimination task. It is also noteworthy that we observed a complex pattern in the dynamics of causal cortical interactions in all task phases, including the anticipatory period, and involving theta, beta and gamma bands. Bastos *et al*.^79^ showed that between visual cortical areas, bottom-up signaling utilizes theta and gamma rhythms, and top-down signaling utilizes the beta rhythm, but exhibit task-dependent dynamic changes. Our data also showed an intense interchange between frequencies related to animal behavior. Indeed, the wide diversity in causal cortical interactions found in our results may be due to the fact that we investigated the information flow in hierarchically distant cortical areas. In addition, the tactile task discrimination employed in this study requires that animals actively explore the behavioral box, which involves complex neural processing, including building expectations, anticipating the task onset, sensory-motor integration, as well as decision making. Our data suggest that all these processes can only be performed properly by the amalgamation of vast territories of cortical circuitry into a highly synchronized and continuous cortical processing neuronal ensemble.

We used Granger causality to investigate the directed information flow in the frontal-parietal-occipital loop during the active tactile discrimination task. GC analysis reveals statistical dependencies in data but is unable to unequivocally describe the neural circuit that explains observed data. For instance, consider the issue of common modulation input. In behaving animals, neural activity in sensory areas is known to be influenced by diverse task aspects such as movement^80^, reward^81^, and attention^82^. Thus, given a pair A and B of recorded brain regions, a positive GC value indicates that there is an information flow from A to B (i.e. knowing the past of A improves prediction of the future activity of B more than the past of B alone) but GC cannot rule out the hypothesis that a third region is in fact modulating A and B. To tackle this issue, one possible approach is to selectively disrupt one brain area and observe the causal effects in the remaining system^83^. In this way, our results should be interpreted with care. We focused our coherence analysis on LFP recordings. However, it would be interesting that future studies could investigate spiking synchronicity or causal connectivity.

## Methods

### Subjects and active tactile discrimination task

All animal procedures were performed in accordance with the National Institute of Health Guide for the Care and Use of Laboratory Animals and were approved by the AASDAP Ethics Committee (CEUA 01/2013). We used nine adult male Long-Evans rats from the IIN-ELS vivarium (Macaíba, Brazil), weighing 300–350 g at the start of training. Animals were housed individually and maintained on a 12/12 hours light-dark inverted cycle at 22 ± 2 °C. Behavioral experiments were conducted during the dark phase of the animal’s cycle. Two days before training, water was available *ad libitum* for one hour per day to perform a behavioral discrimination task as previously described by Krupa *et al*.^40^.

Rats were trained to discriminate between a wide (85 mm) versus narrow (52 mm) aperture using only their mystacial vibrissae to receive a water reward. The behavioral apparatus consisted of two chambers (discrimination and reward) separated by a sliding central door (Fig. 1A,B). The reward chamber has two nose pokes (left and right) connected to a tube which delivers drops of water (~50 µL) when the animal makes a correct discrimination. The discrimination chamber contains a third nose poke located in front of the bars which move with variable aperture (discrimination bar). The behavioral apparatus is located inside a sound-attenuating and light-proof isolation box to ensure that the animal is receiving only tactile stimuli.

The trial session started when the central door opened, and the rat moved in the direction of the discrimination chamber. In this chamber, rats had to use their vibrissae to touch the discrimination bar, poke their nose in the center nose poke and then go back into the reward chamber to receive a water reward. The rats had to poke their nose into the right nose poke if the aperture was wide, and into the left nose poke when the aperture was narrow. If the trial was incorrect, no reward was delivered. The aperture was randomly chosen by a computer controlled by the MedPC Med Associates computer program and DIG computer interface (MED Associates, Inc., St. Albans, VT). The rats were trained until at least 70% of the trials were correct in two consecutive sessions. The duration of each training session was 90 min. For detailed apparatus and task description, see Krupa *et al*.^40^.

For purposes of electrophysiological analysis, the present task was divided in four different phases: anticipatory ([−1.0 −0.4] seconds), discrimination 1 ([−0.4 0] seconds), discrimination 2 ([0 0.4] seconds), behavioral response ([0.4 2.0] seconds), and reward ([2.0 4.0] seconds). Note that the discrimination was divided into two periods, 1 and 2. Discrimination 1 corresponds to the period between −0.4 and 0 s, when the animal passes between the tactile stimulation bars towards the CNP (Central Nose Poke) (t = 0 s); discrimination 2 corresponds to the period from 0 to 0.4 s, when the animal returns from the CNP, passes between the tactile stimulation bars and moves towards the reward chamber (Fig. 1A,B).

### Multi-site, multi-electrode cortical implants

After the initial behavioral training, animals were surgically implanted with microwire arrays in multiples cortical areas. Detailed procedures for chronic array implant are described in Wiest *et al*.^84^. Under deep ketamine (70 mg/kg i.p.) and xylazine (3 mg/kg i.m.) anesthesia, 64 tungsten microwires (diameter of 50 µm) distributed in different arrays were surgically positioned within ACC, PPC, S1 and V1 (Fig. 1C). The number of microwires and the arrangement in each array was as follows: ACC (2 × 8 microwires, spaced 200 µm), PPC (4 × 4 microwires, spaced 300 µm), S1 and V1 (4 × 4 microwires, spaced 400 µm). The following coordinates relative to bregma in millimeters^85^ were used to center the arrays: ACC (+2.06 anteroposterior [AP], +1.40 mediolateral [ML], 1.80 dorsoventral [DV]), PPC (−3.66 AP, 2.55 ML, 0.80 DV), S1 (−2.64 AP, 5.95 ML, 1.20 DV), V1 (–6.20 AP, +4.20 ML, 1.0 DV). After the surgery implant, the microelectrode arrays were fixed with epoxy paste, soldered in a printed circuit board and connected to a miniature connector (Omnetics Connector Corporation, Minneapolis, MN).

### Electrophysiological recordings

Signal acquisition was performed using a Omniplex D Neural Data Acquisition System of 64 channels (Plexon Neurotechnology Research Systems, Dallas, TX) as previously described by Wiest *et al*.^84^. Data were recorded, digitized and stored as spike timestamps and local field potentials (LFP). LFP were preamplified (1000X), filtered (0.3–400 Hz), and digitized at 1000 Hz using a digital acquisition card (National Instruments Corporation, Austin, TX). Spike signals were differentially amplified (20,000–32,000 X), filtered (400 Hz–5 kHz) and digitized at 40 kHz. Spikes from each electrode were classified on-line (Sort Client, Plexon) and off-line using spike-sorting software (Offline Sorter, Plexon Inc., Dallas, TX). For example, see Supplementary Material Fig. S1. Classified signals were analyzed considering the following criteria: inter-spike interval greater than 1.0 ms and waveform shapes stereotypy using principal component analysis and waveform inspection.

### Histology

At the end of the last recording session, all rats were given ketamine (100 mg/kg i.p.) and xylazine (10 mg/kg i.m.) to induce deep anesthesia. These rats were then transcardially perfused with heparin (1 U/ml) in saline (0.9%), followed by 0.1 M phosphate buffered paraformaldehyde (4%, pH 7.4). The brains were removed and stored overnight in phosphate buffered paraformaldehyde at 4 °C. Brains were then transferred into cryoprotection solution (30% sucrose) for 24 hours. Microelectrode locations were histologically verified and confirmed in 50 micron brain slices stained by cytochrome c oxidase staining in (See Supplementary Material Fig. S2).

### Electrophysiological data preprocessing

Neuronal data from 9 rats were processed and analyzed using NeuroExplorer (version 4; NEX Technologies) and custom MATLAB scripts (The MathWorks, Natick, MA). The average number of trials performed per session was 217 ± 86. A trial was defined as the [−4.0 4.0] s period centered on the nose poker beam breaking.

For all LFP data analyses, we first removed any 60 Hz power line noise and harmonics using EEGLAB plugin “cleanline”^86^, which employs a multi-taper regression method that alleviates phase distortion and band holes present on the more common notch filters. Each data channel was z-scored and trials exhibiting anomalous amplitudes (more than 5 standard deviations above mean) were removed. Data was high-pass filtered (cutoff frequency 1 Hz, zero-phase FIR filter) for Granger causality analysis. Lastly, the dataset was resampled to 500 Hz.

### Peri-stimulus time histograms

Peri-stimulus time histograms (PSTH) of neuronal responses were constructed for every single cortical neuron identified in each subject. Neuronal data was binned using a sliding 10 ms time-window, smoothed using a 5-point moving average filter, and a method based on cumulative summed spike counts^43,44^ was used to assess significant deviations from baseline neural activity, defined as the [−2 −1] second period. The method proposed by Wiest *et al*.^87^ is based on bootstrap and identifies the post-baseline period bin in which neuronal activity is in the 1st or 99th percentile of the baseline distribution, thus indicating a significant modulation.

### Type of firing modulations

Individual neuronal activity from each region based on the PSTH analysis was labeled as “increased” or “decreased”, if the neuronal firing rate increased or decreased, respectively, considering the baseline period, or “multiphasic”, if a given neuron showed both increased and decreased modulation. If no modulation was found, neurons were labelled “unresponsive”. In addition to the fraction of each neuron response type, we provide the firing response magnitude (the average difference in firing rate between the significant firing modulation period and the baseline) and the firing response duration (the average time for which the significant firing modulation was sustained)”.

### Phase-locking value

Following Lachaux *et al*.^41^, phase-locking value (PLV) analysis was used to assess pairwise synchrony between LFPs from each recorded region. We first iteratively band-pass filtered the epoched data from each region in sub-bands in the range [3–125] Hz with a frequency bandwidth of 2 Hz. Then, we applied a Hilbert transform for each frequency sub-band to obtain the instantaneous phase of each time series. For a given pair of time series a and b comprised of n samples, PLV at time t was given by:

$$PL{V}_{a,b}(t)=\frac{1}{N}|\sum _{n=1}^{N}\,{e}^{j}({\O }_{a}(t,n)-{\O }_{b}(t,n))|$$

where N is the total number of trials, 𝜙_a_(t, n) − 𝜙_b_(t, n) is the phase difference between time series at time t and trial n, and j is the imaginary unit (See Supplementary Material Fig. S3).

PLV values range from 0 (no phase synchrony) to 1 (perfect phase-locking). To avoid border effects, we discarded the PLV at the first and last 0.5 s of the analysis. In order to assess the influence of background LFP fluctuations on the PLV measure, we randomly shuffled the trials of one time series and calculated PLV 100 times, obtaining an average PLV that would be expected by chance and could thus be used for bias correction. However, as this bias correction did not qualitatively alter the results, the results described in this paper refer to the original PLV calculation standard scored by the baseline (−3 s to −1 s) of each frequency. We considered as significant values those higher than baseline mean plus two times its standard deviation. The spectral PLV maps were smoothed with a Gaussian filter (mask size = [5 350], standard deviation sigma = 50).

### Granger Causality

We employed Granger causality (GC) to characterize causal interactions among LFP time series from the recorded regions. GC is based on the Wiener-Granger concept of causality^61^. First, if knowledge of the past of a time series A improves the prediction of the future of another time series B better than what could be accomplished by knowing the past of B alone, then signal A “Granger-causes” signal B. This statistical concept is increasingly popular in the neuroscience community and offers a directional measure of neural functional connectivity. We used the multivariate Granger causality (MVGC) Matlab toolbox provided by Barnett and Seth^44^ to calculate the multivariate conditional GC in the frequency domain over time, which is more appropriate for neural time series.

First, we fit a multivariate autoregressive (MVAR) model to the LFP data from the 4 regions studied in all trials, using the ordinary least squares method with model order estimated by the Akaike Information Criterion (limited to 20). Next, we obtained a MVAR model and calculated the autocovariance sequence for each sliding window (150 ms length, 10 ms step), which was then used to calculate the pairwise-conditional frequency-domain MVGC. The GC results was standard scored by the baseline (−3 s to −1 s) of each frequency. We considered as significant values those higher than baseline mean plus two times its standard deviation. The spectral GC maps were smoothed with a Gaussian filter (mask size = [20 70], standard deviation sigma = 30).

## Supplementary information

Supplementary information

## Data Availability

The authors states that are available to make materials, data and associated protocols promptly available to readers.
