# Supplementary material for: Frequency-specific coupling in fronto-parieto-occipital cortical circuits underlie active tactile discrimination
Source: Sci Rep. 2019 Mar 25;9:5105. doi: 10.1038/s41598-019-41516-3 (PMC6434051; doi:10.1038/s41598-019-41516-3)
Supplement: Supplementary file 1 — Supplementary information [file 41598_2019_41516_MOESM1_ESM.pdf]

Supplementary Information

FREQUENCY-SPECIFIC COUPLING IN FRONTO-PARIETO-OCCIPITAL CORTICAL CIRCUITS UNDERLIE ACTIVE TACTILE DISCRIMINATION

Carolina Kunicki[1*], Renan Moioli[1], Miguel Pais-Vieira[2,3], Andre Peres[1], Edgard Morya[1], Miguel Nicolelis[1,4,5,6,7]

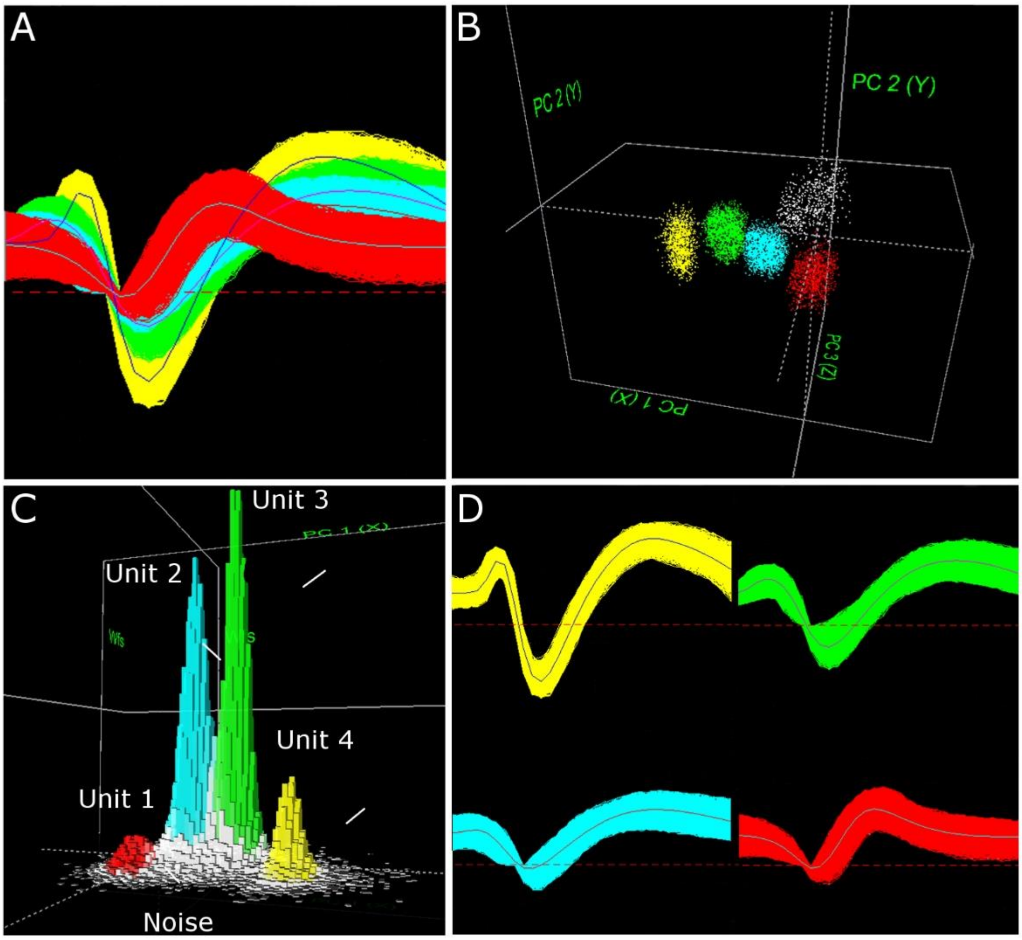

Figure S1: Example of spike sorting from extracellular recording. (A) Superposition of all waveforms recorded from one electrode in ACC. Each color represents a unit (red, green, blue and yellow). (B) Cluster representation in 3D principal component space. (C) Waveform histograms for each unit. (D) Waveforms isolated from the same data presented in Fig. 2A.

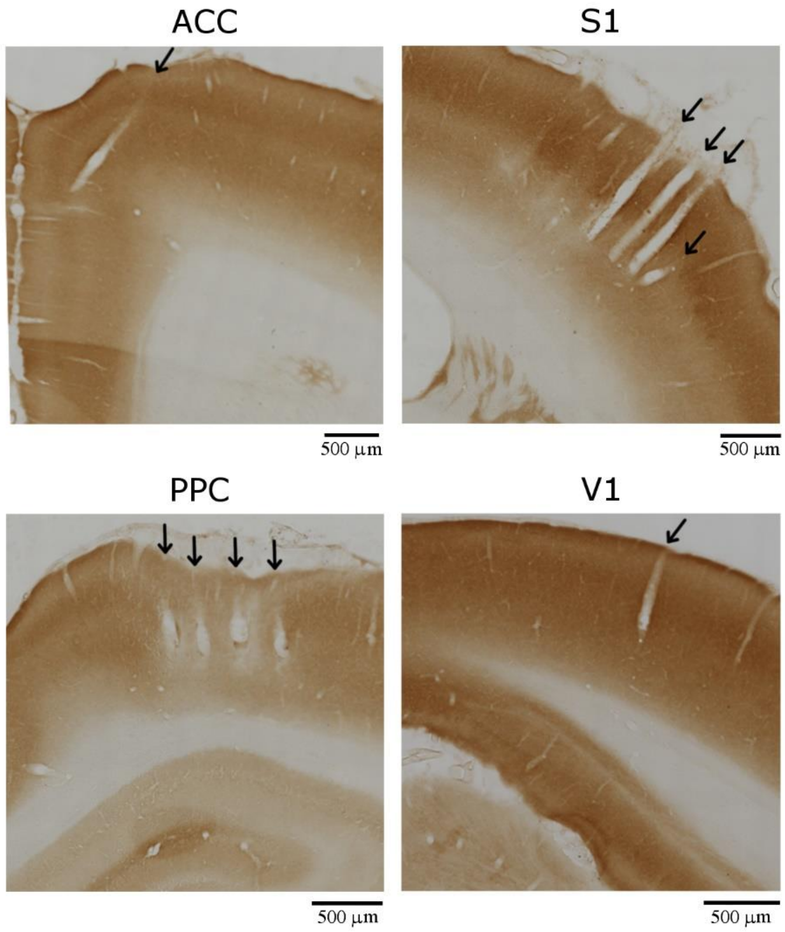

Figure S2: Histological analysis of microelectrodes placement marked by the cytochrome oxidase stain. The image shows 1 electrode track in ACC (bregma 2.76 mm, dorsoventral [DV] 1.80 mm), 4 tracks in S1 (bregma -1.80mm, DV 1.20 mm), 4 tracks in PPC (bregma -3.24 mm, 0.90 mm DV) and 1 track in V1 (bregma -6.96 mm, DV 0.9 mm). Black arrows indicate the site of entry into the cortex.

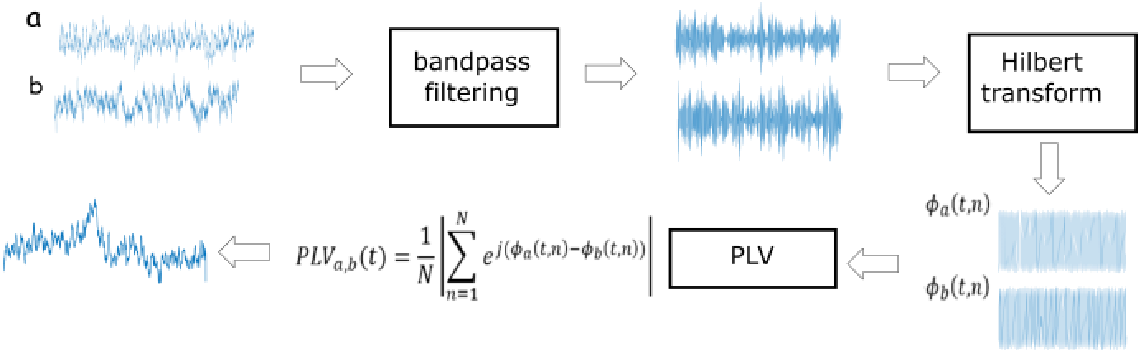

Figure S3: PLV analysis schematics. Following Lachaux *et al.*[1], for a given pair of brain regions *a* and *b*, raw epoched LFP were iteratively band-pass filtered in the range [3 125] Hz with a frequency bandwidth of 2 Hz. Then, the instantaneous phase of each filtered time series sub-band was obtained by a Hilbert transform. Note that phase values range from 0 to 2π. The phase time series was used to calculate PLV, following Equation 1. PLV values range from 0 (no phase synchrony) to 1 (perfect phase-locking). To avoid border effects, we discarded the PLV at the first and last 0.5 s of the analysis.

References

1. Lachaux, J. P., Rodriguez, E., Martinerie, J. & Varela, F. J. Measuring phase synchrony in brain signals. *Hum. Brain Mapp.* **8,** 194–208 (1999).
